# DdrR acts as a DNA damage-induced cell division inhibitor in *Acinetobacter baumannii*

**DOI:** 10.1128/jb.00416-26

**Published:** 2026-09-09

**Authors:** Christian P. Lowe, Deborah Cook, Lily Hoard, McKinnley Martin, Janelle M. Hare

**Affiliations:** 1 Department of Biology and Chemistry, Morehead State University 167097, Morehead, Kentucky, USA; Dartmouth College Geisel School of Medicine, Hanover, New Hampshire, USA

**Keywords:** *Acinetobacter*, DNA damage checkpoints, cell division, UmuDAb, cell division inhibitor

## Abstract

The genus *Acinetobacter* possesses non-canonical genes and responses to DNA damage. It encodes no LexA homolog to repress the ~150 SOS genes induced after exposure to various DNA-damaging agents, but two SOS regulators, UmuDAb and DdrR, repress both themselves and error-prone polymerases that generate mutations after DNA damage. Further differences include the lack of the Gram-negative cell division inhibitor (CDI), SulA, in members of the *Moraxellaceae* family to which *Acinetobacter* belongs. However, *ddrR*, which lacks homologs outside the *Acinetobacter* genus, is transcribed divergently from *umuDAb*, like small CDIs of Gram-positive species lacking SulA, leading us to explore the possibility that DdrR inhibited cell division after DNA damage in the opportunistic *Acinetobacter baumannii*. Like *lexA* mutants of *Escherichia coli* and *Caulobacter crescentus* that overexpress CDIs SulA and SidA, respectively, a null *umuDAb* mutant that overexpresses *ddrR* formed filaments in the absence of DNA damage and further increased its cell length after DNA damage. A strain expressing a non-cleavable UmuDAb showed neither of these phenotypes, implicating a UmuDAb-repressed gene in the inhibition of cell division. However, Δ*umuDAb* Δ*ddrR* double mutant cells regained a wild-type cell length and did not form filaments after DNA damage, and *ddrR* mutant cells had an impaired filamentation response to DNA damage relative to wild type. Conversely, wild-type cells overexpressing *ddrR* became filamented in the absence of DNA damage. *umuDAb* mutant cells overexpressing *ddrR* exhibited impaired growth as measured by increased doubling times, lower optical density, and reduced cell viability. The rescue of these growth impairments by *ddrR* mutation suggested that DdrR functions as a CDI in the DNA damage response of *Acinetobacter*.

**IMPORTANCE:**

In hospital environments, the multi-drug-resistant opportunistic pathogen *Acinetobacter baumannii* incurs DNA damage from antibiotics, UV exposure, and desiccation, which induces multiple error-prone polymerases that can cause clinically relevant antibiotic resistance. However, while *Acinetobacter* species lack the common DNA damage response proteins LexA and SulA, they also show the typical DNA damage response of a cell division checkpoint. We found that the small SOS protein DdrR inhibited cell division like Gram-positive cell division inhibitor (CDI) proteins. After DNA damage, DdrR was required for the cell filamentation response, while increased DdrR expression impaired cell growth and viability. Uncovering a novel mechanism of controlling growth inhibition after DNA damage presents an opportunity to define a new model of inhibiting cell division for non-*Enterobacteriaceae* Gram-negative Gammaproteobacteria.

## INTRODUCTION

Cells use transcriptional and post-transcriptional mechanisms to regulate their responses to DNA damage (1), and these responses can interfere with cell division and cause mutations that drive antibiotic resistance (2, 3). In models originally developed in the Gram-negative *Escherichia coli* (4) and Gram-positive *Bacillus subtilis* (5, 6) bacteria, the DNA damage response (DDR) regulator LexA (DinR in *Bacillus* species) represses a large regulon of SOS genes (7) that conduct DNA repair and recombination (8). Later-induced SOS proteins (9) wield the double-edged sword of error-prone, translesion DNA replication (10, 11) that causes SOS mutagenesis (12, 13). This necessitates multiple mechanisms to maintain the SOS regulon’s tight temporal control (14–16). Yet other DDR genes function to halt cell division until DNA damage is repaired, resulting in cell filamentation (17–19). SOS genes responsible for this cell division checkpoint encode proteins such as SulA, which binds to the septal Z-ring protein FtsZ and inhibits its polymerization and thus cell division (19, 20).

Many modifications to the extensive knowledge built from these canonical model systems exist across bacterial phyla (21). For example, most genera in the Gram-negative *Moraxellaceae* family, such as *Acinetobacter*, lack not only *sulA* (22, 23) but also *lexA* (23, 24). Because the regulation of cell division after DNA damage in this genus is therefore atypical, one species, *Acinetobacter baumannii*, has emerged as a model for DDR studies (24–29). *A. baumannii* is a nosocomial, opportunistic non-enteric pathogen with increasing multi-drug resistance that is categorized as one of the ESKAPE pathogens that are urgent threats to human health (30). It survives desiccation and other environmental stressors in the hospital environment (31–33), and its DDR includes a robust SOS mutagenesis response (24, 29) that contributes to developing antibiotic resistance (29, 34, 35). *A. baumannii* responds to DNA damage by inducing over 100 genes (34, 35) and displays the hallmark cell filamentation of a cell division checkpoint (36). We found that the error-prone polymerases that conduct SOS mutagenesis in *A. baumannii* are co-repressed by the DNA damage-inducible, divergently transcribed, and auto-repressing (37) UmuDAb and DdrR proteins (14, 27, 38–40). However, UmuDAb does not fully replace LexA in the DDR, regulating only ~18% of its DNA damage-induced genes (34), and possessing only 37% identity to LexA (37).

As another example of this DDR diversity, although enteric Gram-negative bacteria in the Class Gammaproteobacteria encode the checkpoint inhibitor SulA (41), SulA is not encoded in the Gram-negative α-proteobacterium *Caulobacter crescentus* (42), nor in several Gram-positive genera (*Bacillus*, *Listeria*, and *Mycobacterium*), including the multi-drug resistant ESKAPE pathogen *Staphylococcus aureus* (43). These bacteria instead enforce DDR checkpoints with cell division inhibitor (CDI) proteins, such as *Bacillus* species’ YneA (18), *C. crescentus* SidA (42), and *S. aureus* SosA (44). These small membrane proteins of 29–165 AA lack homology to other proteins and are transcribed divergently from, but adjacent to, an SOS response repressor (18, 43–48). Crucially, as these CDIs of different species typically also lack homology with each other (43), more of these effectors are likely undiscovered. Due to DdrR’s similarly small size (~71–78 AA), lack of homology outside *Acinetobacter*, and its similar position divergently encoded from the DDR repressor *umuDAb*, we hypothesized that DdrR might similarly control the inhibition of cell division after DNA damage.

This inhibition of cell division is a crucial part of cell responses to DNA damage, as without a pause in cell division after DNA damage, cells can divide while maintaining mutations or damaged DNA bases that may preclude subsequent division. Interfering with divisome components to either cause an increased mutation rate (49) or inhibit the division process to produce a type of cell cycle arrest (50) could inhibit the function and growth of pathogens that are resistant to antibiotics. Our objectives in these experiments were to test whether DdrR had a role in cell division inhibition. We constructed mutant strains either lacking or overexpressing *ddrR* and measured these strains’ morphological and cell growth parameters. Mutants in *ddrR* showed DNA damage-related phenotypes shared by the checkpoint CDIs: growth sensitivity after DNA damage and the inability to halt cell division after DNA damage, as evidenced by cell length changes. Conversely, *ddrR* overexpression in the absence of DNA damage caused cell filamentation, an impaired ability to form colonies, and delayed growth.

## MATERIALS AND METHODS

### Media and growth conditions

*Acinetobacter baumannii* ATCC 17978 (wild type; WT) and derived mutant strains (Table 1) were grown at 37°C on LB (Miller) agar supplemented with antibiotics as appropriate (kanamycin [KAN] at 30 μg/mL, gentamicin [GEN] at 20 μg/mL, ampicillin [AMP] at 100 μg/mL, and streptomycin [STR] and spectinomycin [SPT] at 40 μg/mL). Liquid cultures were grown at 37°C, shaking at 225 rpm in LB (Miller) or minimal media (37) supplemented with 10 mM sodium succinate with appropriate antibiotics. Arabinose for protein expression was added at indicated concentrations of 0.1%–1%, and mitomycin C (MMC) for DNA damage at 2 μg/mL as needed.

**TABLE 1:** Strains used in this study

Strain name	Genotype; description	Reference
17978	*A. baumannii* ATCC 17978; wild type	ATCC (51)
JH1600	17978 Δ*umuDAb*::*kanR*; KAN^R^	(34)
JH1700	17978 *ddrR*::Tn*LK*; *lacZ-kanR* inserted into codon 16, KAN^R^	(38)
BC1	17978 *umuDAb A83Y*	(14)
JH1173	17978 Δ*A1S_1173 rumB::Ω*; STR^R^ SPT^R^	(34)
JH636	17978 Δ*A1S_0636 umuD::kanR*; KAN^R^	(34)
MF2	17978 *umuDAb K40P N41P* (HTH2 mutation)	(14)
DC5	17978 *ddrR E14X*; ochre nonsense mutant	This study
DC8	17978 *umuDAb* 1_17*del ddrR* 1_195*del*	This study
DC11	17978 *ddrR1p* (*ddrR-umuDAb* operator mutation “C1” [27])	This study

### Plasmid and mutant strain constructions

Recombinant plasmids expressing DdrR or UmuDAb from pVRL2 were made by restriction and insertion cloning. 5′ primers containing a *Xho*I restriction site (DdrRXho5′, DdrRlongXho, and UmuDAbXhoI) and 3′ primers with a *Xba*I restriction site (DRstopXba and UDstopXba) were used to amplify *A. baumannii* 17978 genes of interest (Table 2). PCR amplicons and the pVRL2 vector were digested with these same enzymes, then ligated to generate expression plasmids, which were confirmed with DNA sequencing.

**TABLE 2:** Primers used in this study

Primer name	Sequence	Reference
UmuDAbXhoI	ATCGCTCGAGATGCCAAAGAAGAAAGAATTC	(27)
UDstopXba	AATTTATCTAGATTATCTCATTCGTTTGAG	This study
DdrRXho5′	ATAATCTCGAGATGAAAAATCAACGTGATGC	This study
DdrRlongXho	ATAATCTCGAGTCATGATCAGATTTTAATCCATTTTGC	This study
DRstopXba	AATTTATCTAGATTATGAGTGGGTAAGGGG	This study
UDPromPstFor2	CGTCAACTTGAAACTGCAGCTTTTTCAAGTTACATTGTTTGTGTAGG	This study
UDPromPstRev2	CCTACACAAACAATGTAACTTGAAAAAGCTGCAGTTTCAAGTTGACG	This study
17978stopddrRFor	GGCATGTTGTTAATCTGCATAAC	This study
17978stopddrRRev	GTTATGCAGATTAACAACATGCC	This study

Mutant 17978 strains were made by performing Quick Change II XL site-directed mutagenesis (Agilent) or restriction site-based mutations on pEX18Gm suicide vectors (52) containing 17978 genomic DNA. After DNA sequencing confirmation of the mutations, plasmids (see Table 3) were electroporated into 17978 cells for recombination into the chromosome. Allelic replacement mutants were further confirmed by DNA sequencing of PCR amplification products from the chromosome. DC5 and DC11 are unmarked by any antibiotic resistance cassettes. Specifically, the unmarked *ddrR E14X* ochre nonsense mutant strain DC5 was constructed after performing site-directed mutagenesis with primers 17978stopddrRFor and 17978stopddrRRev on plasmid pEX17M, which contains a ~4 kb genomic fragment containing *umuDAb* and *ddrR* (14). DC11 (*ddrRp1*) bears an unmarked mutation corresponding to the C1 mutation in the core region of the UmuDAb binding site (27). The chromosomal C1 mutation was constructed after performing site-directed mutagenesis with primers UDPromPstFor2 and UDPromPstRev2 on plasmid pEX17M. DC8 was formed by, first, deleting a 492 bp *Eco*RI fragment of p17MK (14) to remove the N-terminal 64 and 7 amino acids of DdrR and UmuDAb, respectively, as well as their promoters and joint UmuDAb binding site. This formed p17MKdRuD. Ligation of a p17MKdRuD *Bam*HI and *Nru*I fragment containing the 17978 genomic region into pEX17M formed pEX17MKdRuD, which was used to construct DC8.

**TABLE 3:** Plasmids used in this study

Name	Description	Reference
pVRL2	Arabinose-inducible expression vector for *Acinetobacter* spp.; GEN^R^	(53)
pUDAb	pVRL2 expressing UmuDAb; GEN^R^	This study
pDR5	pVRL2 expressing DdrR; GEN^R^	This study
pDR5*	pVRL2 expressing DdrR E14X; GEN^R^	This study
p17M	pGEM-T bearing 4 kb 17978 genomic *umuDAb ddrR* locus; AMP^R^	(14)
p17MK	p17M bearing Kan^r^ of pCRII; AMP^R^ KAN^R^	(14)
pEX17M	pEX18Gm bearing 4,092 bp *Sph*I-*Sac*I fragment of p17M; GEN^R^	(14)
pEX18Gm	Suicide vector; GEN^R^	(52)
p17MKdRuD	p17MK bearing 492 bp *Eco*RI fragment deleted; KAN^R^	This study
pEX17MKdRuD	*Bam*HI-*Nru*I fragment of p17MKdRuD ligated into pEX17M; GEN^R^	This study

### Cell length measurements

Overnight cultures were used to make two subculture dilutions. For arabinose and MMC studies, each treatment was immediately added to one of the subcultures, while the other was left untreated as a control, and both were allowed to grow for 4 h. Cells were pelleted and resuspended in 1× phosphate-buffered saline before dispensing on slides, air drying, heat fixation, and crystal violet staining. Micrographs were taken at 1,000× on a Leica ICC50 HD microscope and camera. Cell lengths were measured with MicrobeJ (54), a plug-in for ImageJ (55).

### Cell viability and mass measurements

Cultures were grown in minimal media overnight then normalized to an optical density at 600 nm (OD_600_) of 0.02 and regrown with OD_600_ measured every 30 min until an OD_600_ of ≥0.05 was reached. The Δ*umuDAb* mutant’s growth only reached an OD_600_ of 0.04 in the three trials, so each trial began when the wild-type (WT) and JH1700 (*ddrR*::Tn*LK*) strains’ OD_600_ was 0.05. Cultures were split in half, and 2 μg/mL MMC was added to one culture at time zero (T0). At T0, an aliquot of each culture was taken to prepare serial dilutions for CFU/mL plating, then the cultures were grown for 90 min (T90). The same process was repeated at T90 when final OD_600_ measurements were taken.

### Growth rate measurements

Three overnight LB cultures for each strain were prepared. An aliquot from each sample was diluted twice to an OD_600_ of 0.01. The two dilutions totaled 1 mL each and the same growth medium was used as the diluent. One dilution was left untreated while the other received a dose of MMC. Technical triplicates of 200 µL per well were loaded into a 96-well plate and grown in a BioTek Epoch 2 instrument (Agilent). The BioTek Epoch 2 growing conditions were 37°C with continuous orbital shaking over a period of at least 8 h, with the OD_600_ measured every 10 min. Doubling time was calculated using BioTek Gen6 software formula: 60 × 1,000 × ln(2)/DS2, where DS2 represents the mean of the maximum OD_600_ achieved during the run. The onset of stationary phase was calculated using the formula (DS2 – Y intercept OD_600_)/Max V. The comparison of the non-linear fit of growth to an exponential equation was performed in GraphPad Prism 10.6.1.

## RESULTS

### *umuDAb* mutant cells are filamented but increase cell length further after DNA damage

**Fig 1:**
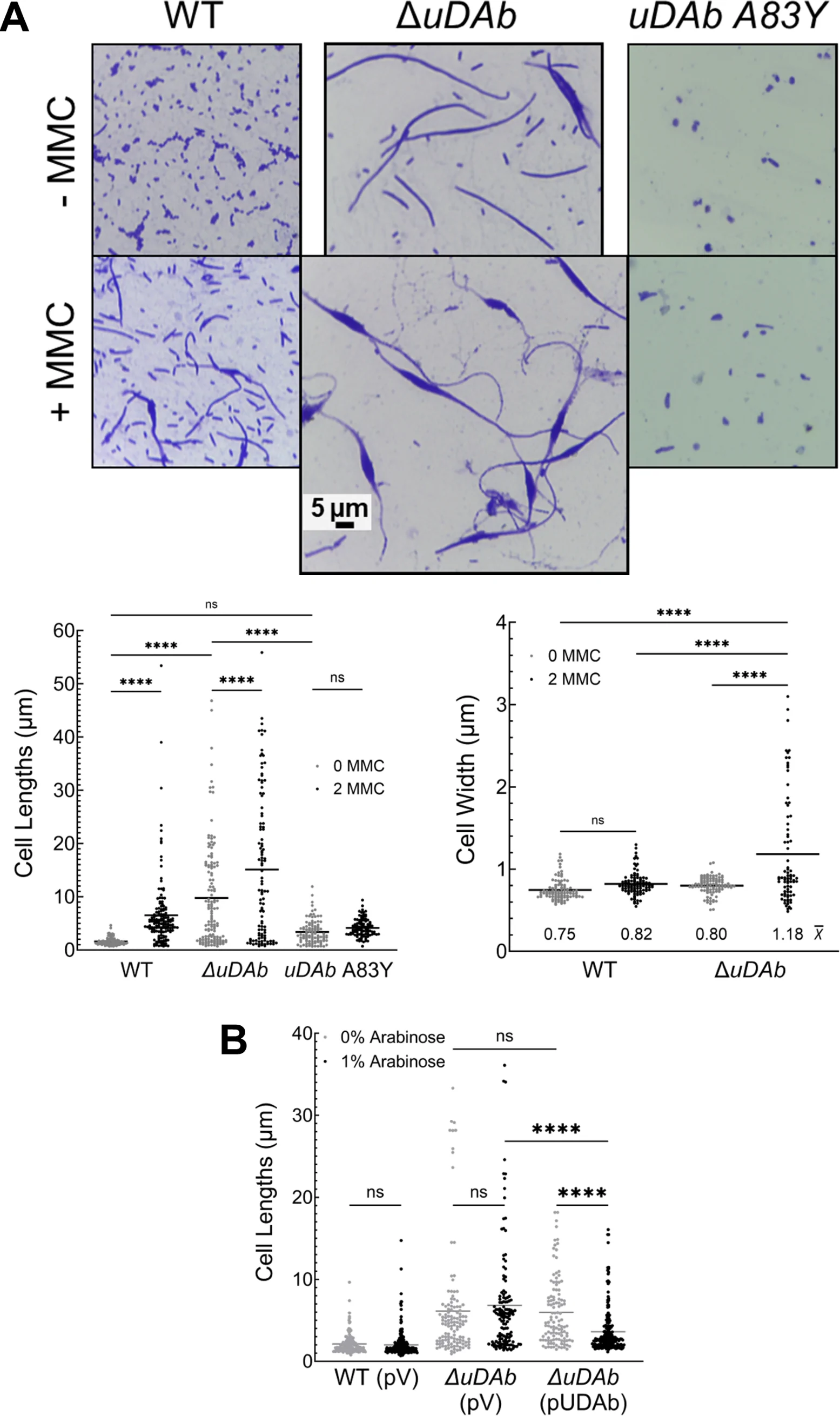
The morphology of cells containing *umuDAb* mutations. The morphology of the WT strain, and strains containing a *umuDAb* mutation (either a null Δ*umuDAb* mutation in strain JH1600 [“Δ*uDAb*”] or a *umuDAb A83Y* self-cleavage mutation in strain BC1 [“*uDAb A83Y*”]) were examined either with or without DNA damage. (**A**) Cell morphologies of WT, Δ*uDAb*, and *uDAb A83Y* in minimal media after 3 h of growth while untreated or exposed to 2 μg/mL MMC, shown in micrographs at 1,000× (on top). Cell lengths (bottom left) and cell widths (bottom right) were quantified from 80 to 151 cells. (**B**) Cell lengths after addition of empty pVRL2 vector (“pV”) to WT and Δ*uDAb* strains, and complementation of the Δ*uDAb* strain by pVRL2 carrying *umuDAb* (“pUDAb”), followed by growth in LB 4 h after addition of 1% arabinose or left untreated. Cell lengths were measured with MicrobeJ after staining with crystal violet. *n* = 115–255 cells in panel **B**. Horizontal bars represent the mean. Pairwise comparison bars represent one-way ANOVA and Šídák’s multiple comparison tests. **** =*P* < 0.03001; *** =*P* < 0.001; ** =*P* < 0.01; * =*P* < 0.05. ns, not significant.

Because UmuDAb shares some biological functions with LexA, notably the repression of several DNA damage-inducible genes (34), we tested whether, like a Δ*lexA* mutant, an *A. baumannii* ATCC 17978 Δ*umuDAb* mutant strain would show an altered morphology in the absence of DNA damage. In such growth conditions, we saw that null Δ*umuDAb* mutant cells (“*uDAb*”), whose entire regulon is de-repressed (34), were elongated compared to wild-type *A. baumannii* 17978 (“WT”) cells (Fig. 1A). However, cells of a *umuDAb A83Y* strain expressing an uncleavable UmuDAb mutant protein, whose regulon is fully repressed (to WT levels [14]), were no different in length than WT cells and shorter than the Δ*uDAb* strain (Fig. 1A). UmuDAb expression from the *Acinetobacter* expression vector pVRL2, corresponding to ~5.5-fold increased expression of beta-galactosidase (53), complemented the Δ*umuDAb* mutant, restoring wild-type cell length cells (Fig. 1B). This complementation showed a dose-dependent effect of shorter cells with increasing amounts of arabinose in the medium (Fig. S1), consistent with previous reports correlating arabinose treatment to expression levels (53). Addition of arabinose to empty vector-containing cells did not produce a significant change in cell length (Fig. 1B).

A well-known bacterial response to various forms of DNA damage and stressors is to halt cell division and form filamented cells; WT cells likewise respond to MMC-induced DNA damage by forming long filamented cells (36). We measured cells in the presence and absence of MMC and observed that both the WT and the already long Δ*umuDAb* cells’ lengths were significantly longer after MMC treatment (ANOVA, *P* < 0.0001) (Fig. 1A). Notably, the Δ*umuDAb* cells also displayed a wider, bulged appearance at mid-cell after MMC treatment (ANOVA, *P* < 0.0001), which did not occur in WT cells. However, the strain expressing uncleavable UmuDAb A83Y (14) did not filament after DNA damage (Fig. 1A). These results implicated a UmuDAb-repressed gene in the filamentation phenotype.

### Aberrant cell length in the Δ*umuDAb* strain disappears after mutation of *ddrR*

Because UmuDAb represses multiple genes in the SOS response of *Acinetobacter* spp. (34, 35), we hypothesized that the Δ*umuDAb* cells’ aberrant length phenotype resulted from derepression of a UmuDAb-repressed gene with CDI properties, similar to LexA repression of the CDI SulA. Additional support for this hypothesis came from seeing that cell lengths were significantly increased in MF2 cells expressing a UmuDAb helix-turn-helix mutant (HTH2) (Fig. S2) that has impaired DNA binding and increases DdrR expression 165-fold in RT-qPCR experiments (14).

**Fig 2:**
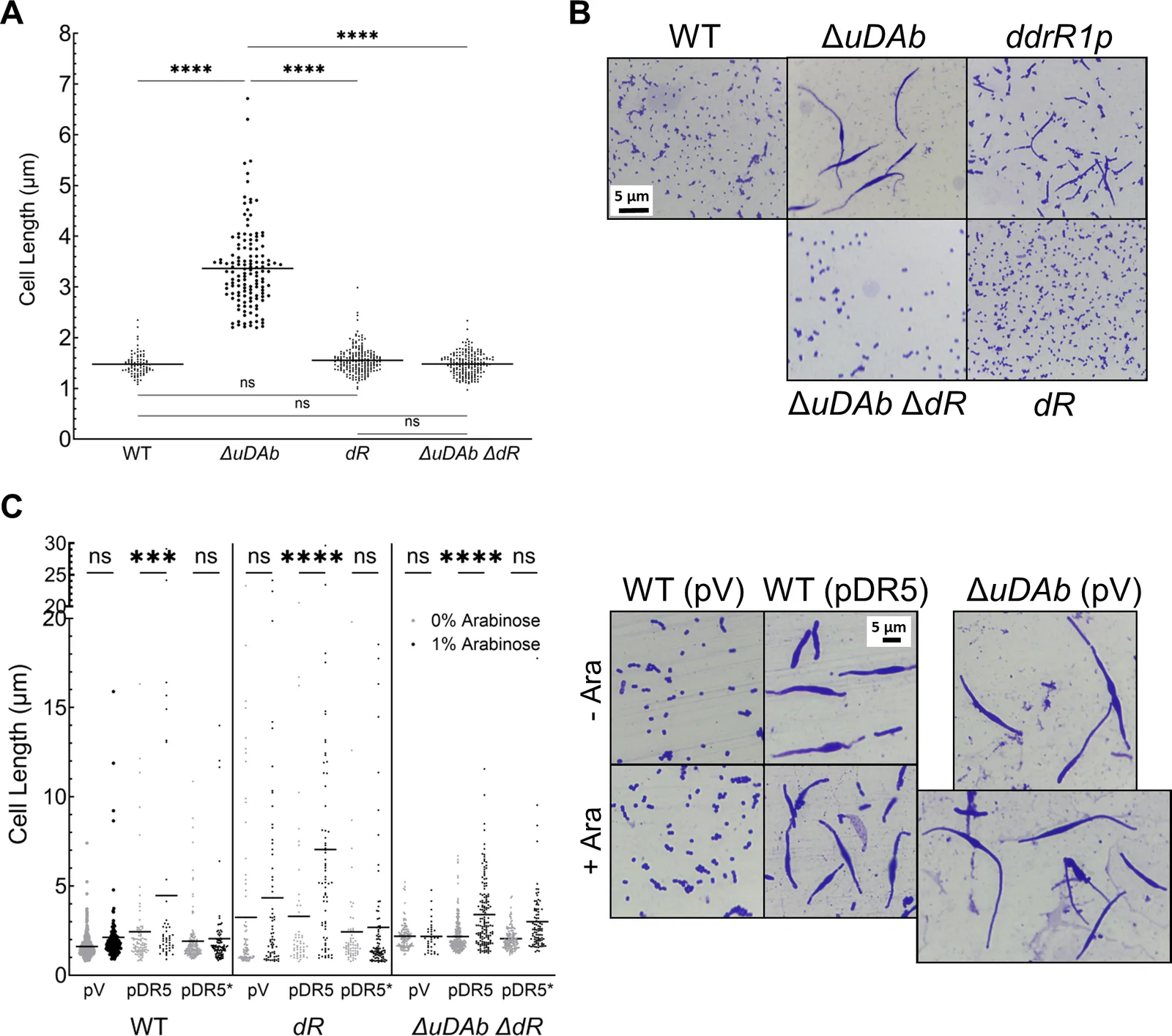
The effects of *ddrR* mutation on cell lengths in the absence of DNA damage. Cell lengths of WT and mutant strains expressing different amounts of DdrR. (**A**) Cell lengths of WT and mutant strains Δ*umuDAb* (”Δ*uDAb*”), *ddrR*::Tn*LK* (“*dR*”), and Δ*umuDAb* Δ*ddrR* (“Δ*uDAb* Δ*dR*”), after 4 h of growth in LB without MMC treatment. (**B**) Micrographs of WT, Δ*uDAb*, *dR*, Δ*uDAb* Δ*dR*, and the *ddrR1p* strain DC11, which bears an unmarked C1 mutation in the shared UmuDAb binding site regulating *ddrR* and *umuDAb* transcription (27). (**C**) Cell lengths of WT, *dR*, and Δ*uDAb* Δ*dR* strains with either empty vector pVRL2 (“pV”) or a pVRL2-based complementation plasmid expressing *ddrR* (“pDR5”) or *ddrR E14X* (“pDR5*”). Cells were grown for 4 h in LB medium with or without 1% arabinose (“Ara”) to induce expression. Cell lengths were measured with MicrobeJ (*n* = 42–206 [panel A] or *n* = 33–297 [panel B], in each of the panel’s three biological replicates) after staining with crystal violet and micrographs taken at 1,000×. Horizontal bars represent the mean. Pairwise comparison bars represent one-way ANOVA with Šídák’s multiple comparisons test. **** =*P* < 0.0001; *** =*P* < 0.001; ** =*P* < 0.01; * =*P* < 0.05. ns, not significant.

In a Δ*umuDAb* strain, the only genes besides *umuDAb* and *ddrR* (the UmuDAb corepressor) that are expressed at higher levels than in WT cells are the *umuDC* error-prone polymerase genes *A1S*_*2008*, *2015*, *1174-1173*, and *0636-0637* (40). No genes are expressed at lower levels in the absence of UmuDAb (40). As *umuDC* genes have well-studied polymerase functions in *A. baumannii* (56, 57), but *ddrR* has co-regulatory actions, we investigated the possible role of DdrR in CDI. We hypothesized that if DdrR functioned in cell division inhibition, cells under- or over-expressing DdrR might display altered growth phenotypes. Because *ddrR* expression increases ~47-fold in the null Δ*umuDAb* mutant, relative to WT (in RNASeq experiments [34]), we hypothesized that this excess *ddrR* expression caused cell elongation in the Δ*umuDAb* strain. Therefore, we tested whether loss of *ddrR* would reverse the cell morphology defect in Δ*umuDAb* cells. We constructed a double mutant Δ*umuDAb* Δ*ddrR* strain (“Δ*uDAb* Δ*dR*”; DC8) and observed that it regained WT cell lengths (Fig. 2A and B). This suggested that the loss of *ddrR* prevented cell elongation in Δ*umuDAb* cells that overexpress *ddrR*.

### DdrR overexpression causes cell elongation in the absence of DNA damage

We also directly tested whether DdrR functioned as a cell division inhibitor by examining whether *ddrR* overexpression would cause cells to filament in the absence of DNA damage. We measured cell lengths in WT, *ddrR::*Tn*LK* (“*dR*”; JH1700) and Δ*umuDAb* Δ*ddrR* mutant cells, each expressing *ddrR* from the pVRL2 expression vector on plasmid pDR5. The addition of arabinose to these strains containing empty vector did not significantly change cell lengths (Fig. 2C). Consistent with our hypothesis, though, we observed that arabinose-induced *ddrR* expression from pDR5, under induction conditions that yield ~5.5-fold increased expression (53), resulted in significant increases in cell lengths of WT, *ddrR*, and Δ*umuDAb* Δ*ddrR* mutant cells (Fig. 2C). In the WT cells, widening also occurred in the middle of cells, strongly resembling the Δ*umuDAb* phenotype in Fig. 1A and 2C. A plasmid with a nonsense mutation at codon 14 of *ddrR* (pDR5*) did not cause increased cell lengths after arabinose treatment (Fig. 2C), suggesting that the DdrR protein caused the cell elongation.

We also tested how de-repressing expression specifically and only from the *ddrR* and *umuDAb* promoters affected cell length. Our previous gel mobility shift assays showed that a competitor oligonucleotide with a 2 bp point mutation (“C1”) in the shared *ddrR–umuDAb* operator reduced UmuDAb binding by ~40% (27). As this C1 mutation only decreases UmuDAb binding to the *ddrR–umuDAb* operator, but not the *umuDC* operators, this experiment tested whether increased DdrR and UmuDAb expression could affect cell lengths. We constructed an unmarked WT strain carrying this C1 *ddrR1p* mutation (DC11) and observed that it formed significantly longer cells in the absence of DNA damage (Fig. 2B; Fig. S2).

### A *ddrR* mutant cannot mount a normal filamentation response to DNA damage

**Fig 3:**
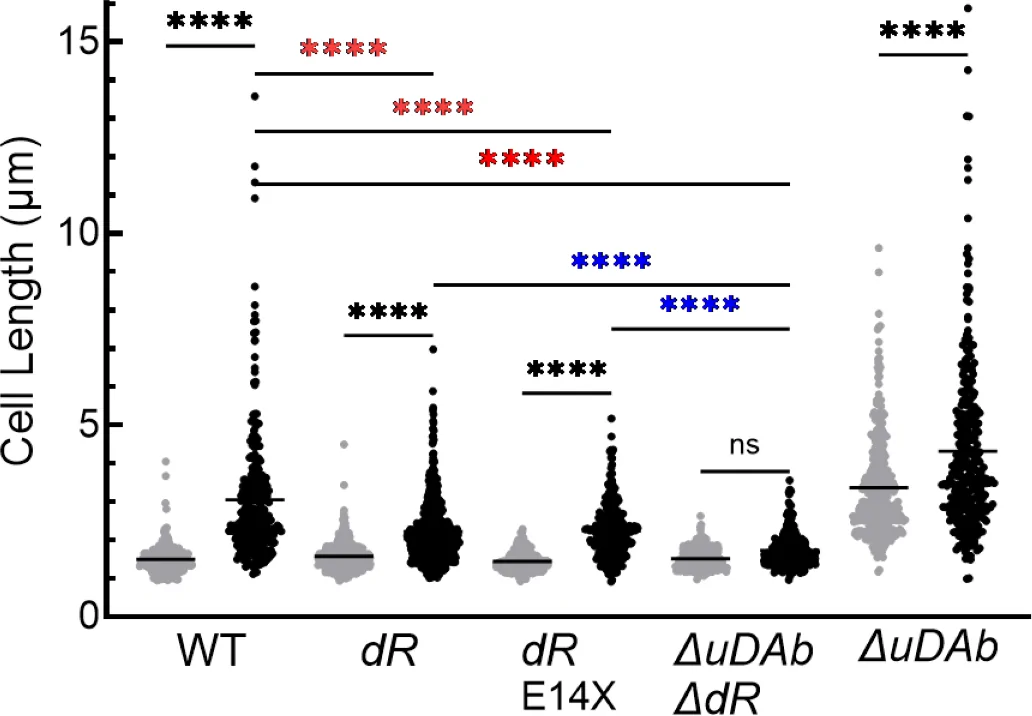
The ability of strains to form cell filaments after DNA damage. Cell lengths were measured for WT and mutant strains *ddrR*::Tn*LK* (“*dR*”), *ddrR E14X* (“*dR E14X*”), Δ*umuDAb* Δ*ddrR* (“Δ*uDAb* Δ*dR*”), and Δ*umuDAb* (“Δ*uDAb*”) that were grown in the presence of (black dots; +) or without (gray dots; −) exposure to 2 μg/mL MMC for 4 h in LB. MicrobeJ was used to measure the length of cells (*n* = 43–323 per each of three biological replicates per strain) after staining with crystal violet. Horizontal bars represent the mean. Pairwise comparisons represent one-way ANOVA with Šídák’s multiple comparisons test. **** =*P* < 0.0001; *** =*P* < 0.001; ** =*P* < 0.01; * =*P* < 0.05. ns, not significant.

The observation that cell filamentation depended on a UmuDAb-repressed gene (Fig. 1A) led us to test whether the UmuDAb-repressed DdrR protein was needed for filamentation after DNA damage. We observed slightly longer cells of the *ddrR::*Tn*LK* insertion mutant (JH1700) after MMC treatment, but neither these cells nor a *ddrR E14X* nonsense mutant (“*dR E14X*”; DC5) displayed cell elongation to the same extent as WT cells after MMC treatment (Fig. 3), suggesting that a DdrR protein was involved in the stress response to MMC that results in cell filamentation. We also measured Δ*umuDAb* Δ*ddrR* mutant cell lengths in response to DNA damage. Unlike the Δ*umuDAb* strain*,* these doubly mutated cells did not elongate after MMC exposure (Fig. 3), again suggesting that *ddrR* was required for the DNA damage response of cell filamentation. Cell lengths did not differ among any of the five tested strains in non-DNA-damaging conditions, with the exception of the Δ*umuDAb* strain.

We next tested whether mutants of the remaining UmuDAb-repressed genes *umuDC* (or *umuDrumB* [34]) that lack polymerase V action would have unimpaired cell filamentation responses. Both Δ*umuD A1S_0636* and Δ*rumB A1S_1173* cell lengths were significantly longer after DNA damage, with cell lengths increasing after MMC treatment by 1.7-fold (WT and Δ*umuD* strains) to 1.9-fold in the Δ*rumB* strain (Fig. S3). Significantly, unlike *ddrR*::Tn*LK* cells, whose untreated cell length was the same as WT cells, but induced cell length was only 63% of the WT, the Δ*rumB* mutant’s cell lengths were consistently ~60% that of WT cells in both untreated and inducing conditions. Neither the Δ*umuD* nor the Δ*rumB* mutant strain had elongated cells in the absence of DNA damage, relative to WT cells (Fig. S3). These experiments indicated that although these genes were regulated by UmuDAb, their loss did not confer cell division defects.

### DdrR expression alters growth of cells

**Fig 4:**
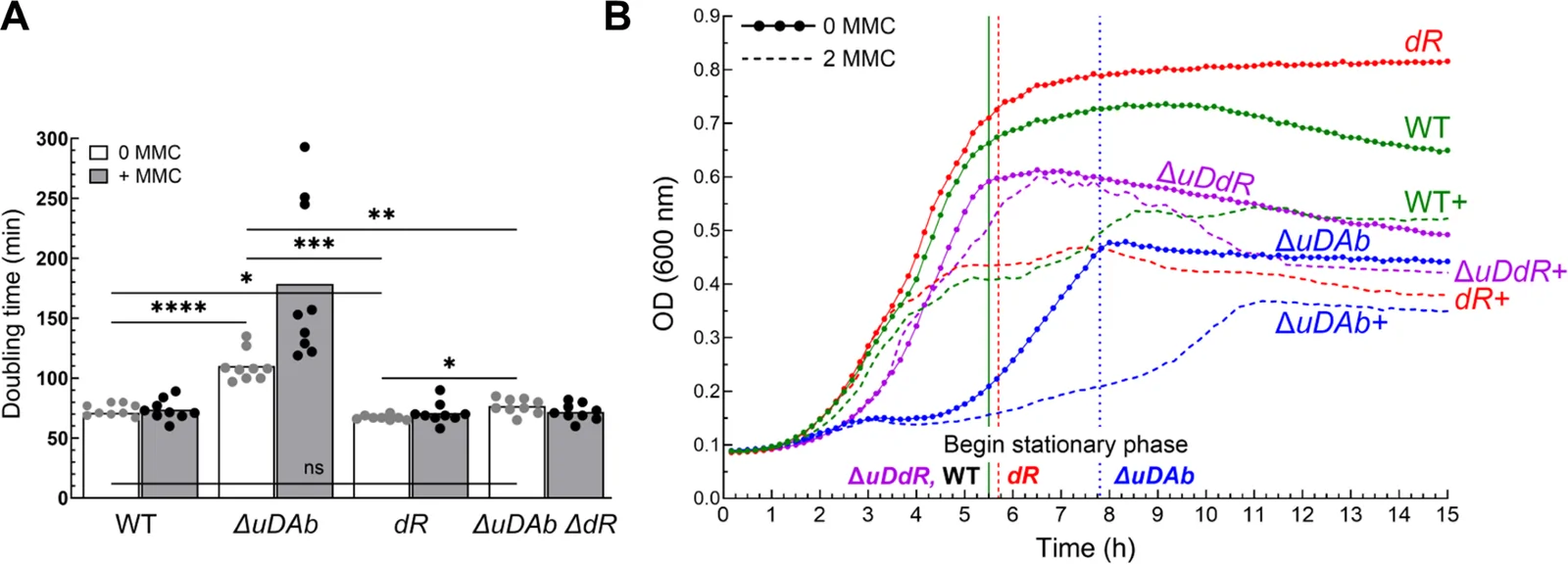
Analysis of population growth parameters. Overnight minimal medium cultures were diluted to an OD_600_ of 0.01, split into two cultures, and one culture was treated with 2 μg/mL MMC. Cultures of WT, Δ*umuDAb* (“Δ*uDAb*”), *ddrR*::Tn*LK* (“*dR*”), and Δ*umuDAb* Δ*ddrR* (“Δ*uDAb* Δ*dR*”) strains were grown in 96-well plates in a BioTek Epoch 2 plate reader with the OD_600_ of cells measured every 10 min. Data are from three independent experiments, each containing technical triplicates of three biological replicates. (**A**) Doubling times in the presence and absence of DNA damage were calculated with BioTek Gen6 software, and ANOVA mixed-effects analysis with Dunnett’s multiple comparison tests was used to compare these doubling times within strains and between untreated strains. Pairwise comparisons are represented by **** =*P* < 0.0001; *** =*P* < 0.001; ** =*P* < 0.01; * =*P* < 0.05. ns, not significant. (**B**) A growth curve of the average of three independent biological cultures; the strain name followed by “+” indicates treatment with MMC. Strain Δ*umuDAb* Δ*ddrR* is abbreviated “Δ*uDdR*” in this panel. The onset of stationary phase calculated with Gen6 software is represented by a solid vertical line for the WT and Δ*uDAb ΔdR* strains, and dashed vertical lines for other strains.

To test the effects of DdrR and UmuDAb expression on cell growth, we measured doubling times and stationary phase onset of WT and mutant strains. The *ddrR*-overexpressing Δ*umuDAb* strain grew significantly slower (110 min doubling time; *P* < 0.01) than the WT (74 min) or *ddrR*::Tn*LK* insertion mutant JH1700 (67 min) (Fig. 4). The Δ*umuDAb* strain also showed significantly slower growth after MMC exposure, as its doubling time increased to 179 min (*P* < 0.01). Conversely, JH1700 cells showed a significantly increased, albeit modest (~10%), growth rate relative to WT, as did the *ddrR E14X* nonsense mutant (“*dR E14X*”; DC5), which showed the same statistically significant results as JH1700 in both Fig. 4A and B (data not shown). Most significantly, further mutating *ddrR* in the Δ*umuDAb* background restored a WT-like doubling time of 77 min to the Δ*umuDAb* Δ*ddrR* strain DC8.

*ddrR* overexpression also impacted other cellular growth dynamics. Notably, the Δ*umuDAb* strain’s growth curve differed from all others (*P* < 0.01 in a comparison of the non-linear fit of growth to an exponential equation) (Fig. 4B). This strain was also significantly delayed (over 2 h) in reaching the stationary phase, relative to all other strains, in both control and MMC-treated conditions (*P* < 0.001 in a two-way ANOVA), and never produced an OD_600_ as high as other strains (Fig. 4B). Both the slower doubling time and delayed onset of stationary phase suggested that *ddrR* overexpression inhibited cell growth.

Consistent with these observations of DdrR inhibiting cell growth, the two *ddrR* mutant strains also reached stationary phase ~20% faster (4.8–4.9 h) than WT (6.2 h) in the presence of DNA damage (two-way ANOVA, *P* < 0.01). Conversely, in the absence of DNA damage, the *dR* mutants’ OD_600_ continued to increase after stationary phase onset, whereas the WT strain displayed a modest death phase (Fig. 4B). Hence, we observed that *ddrR* mutation contributed to cell growth.

### DdrR overexpression impairs colony formation and cell mass increase

**Fig 5:**
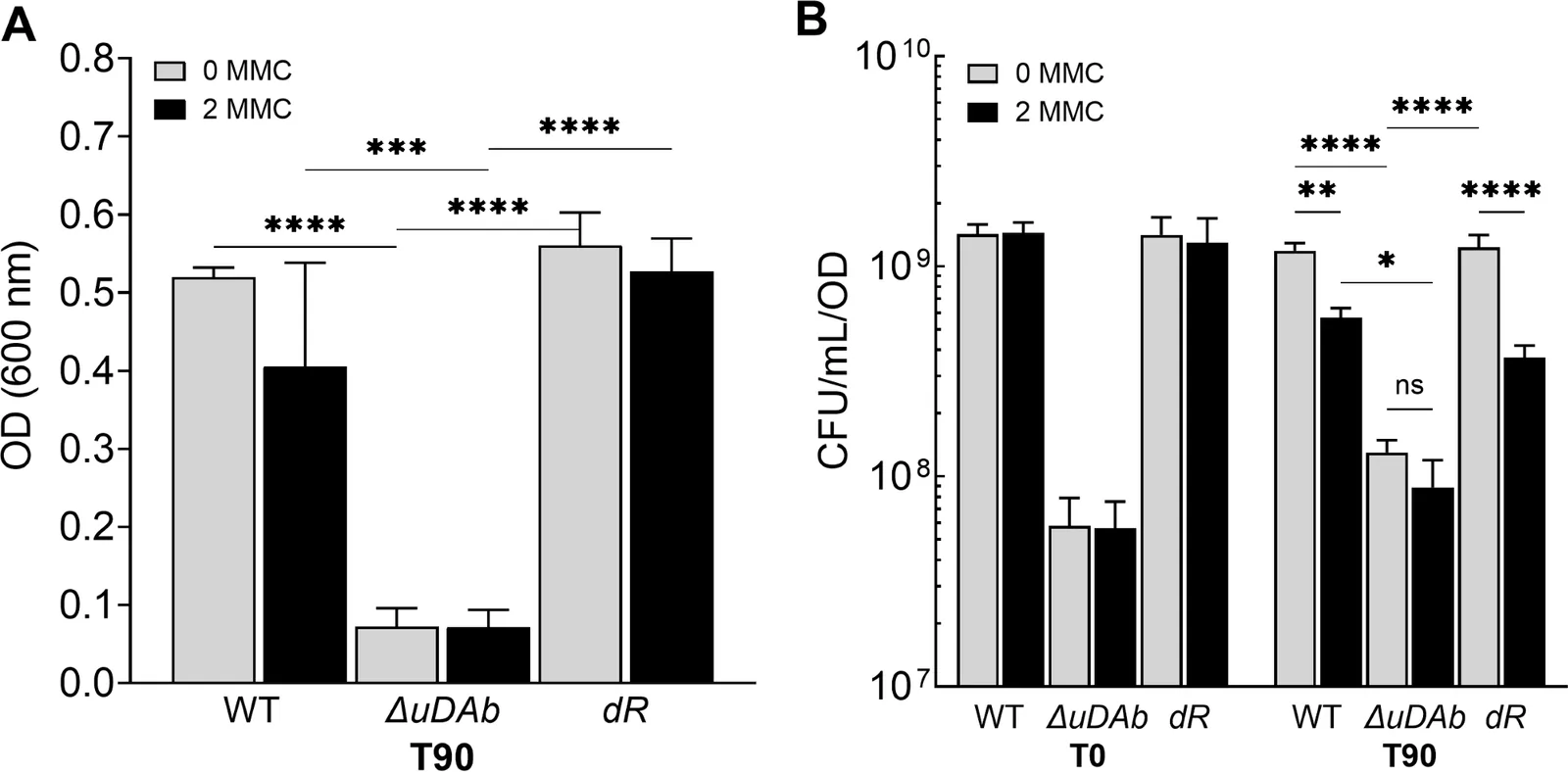
Assays of cell mass and viability. Growth parameters were measured in WT cells and in strains either overexpressing *ddrR* (the Δ*umuDAb* strain “Δ*uDAb*”) or *ddrR*-underexpressing (*ddrR::*Tn*LK*; “*dR*”). Strains were grown in minimal medium with or without 2 μg/mL MMC. (**A**) Strains’ OD_600_ after 90 min growth (T90). (**B**) CFU/mL normalized for OD_600_ values at 0 min (T0) and T90 (there was no difference at T0 between treatments of samples within the same strain). Pairwise comparison bars represent repeated measures two-way ANOVA with Tukey’s multiple comparisons post-testing. Three biological replicates are represented. **** =*P* < 0.0001; *** =*P* < 0.001; ** =*P* < 0.01; * =*P* < 0.05. ns, not significant.

We also evaluated cell viability and cell mass accumulation (via OD_600_) in WT, *ddrR*-deficient, and *ddrR-*overexpressing strains. As a control, we measured the CFU/mL of each strain before the beginning of the experiment (T0 in Fig. 5B), which was the same in the treated and untreated cultures. After 90 min of growth, however, both the colony counts and OD_600_ measurements were lower in the *ddrR-*overexpressing strain (“Δ*uDAb*”; JH1600, in both DNA-damaging and control conditions, than in WT and the *ddrR*::Tn*LK* mutant strain (“*dR*”; Fig. 5A). Furthermore, cell viability was lower in the Δ*uDAb* mutant strain even after normalizing CFU/mL values to cell mass (OD_600_) in Fig. 5B. There was no difference between the OD_600_ values of the WT and *ddrR*::Tn*LK* mutant cultures, in either the MMC-treated or untreated conditions. The *ddrR*::Tn*LK* strain, however, had significantly lower cell viability than WT after MMC treatment (30% vs 48%, respectively; *P* < 0.05 in a *t*-test from four independent experiments). Conversely, the *ddrR* overexpressing Δ*uDAb* strain, JH1600, was significantly less affected by MMC treatment (72% survival) than WT cells. Taken together, these data suggested a role for DdrR expression in maintaining cell viability after DNA damage.

## DISCUSSION

In this paper, we present evidence that, like several Gram-positive bacteria (44, 45), a Gram-negative pathogen also encodes a CDI adjacent to its DNA damage repressor (UmuDAb [34], as *Acinetobacter* species do not encode LexA [22, 23]). We tested WT and mutant strains for known CDI phenotypes after *ddrR* under-expression and overexpression conditions (under basal conditions, the Δ*umuDAb* mutant strain expresses over fourfold higher levels of *ddrR* than WT cells even after MMC treatment [40]). These experiments uncovered multiple phenotypes of DdrR in controlling cell division, both before and after DNA damage, that are also seen in Gram-positive CDIs (44). These included growth sensitivity of a *ddrR* mutant (Fig. 5) and inhibition of cell division after DNA damage (Fig. 3), cell elongation dependent on an intact *ddrR* open reading frame (Fig. 2), and decreased colony viability and cell growth after *ddrR* overexpression (Fig. 5). Strikingly, mutating *ddrR* in *ddrR*-overexpressing cells restored a WT-like cell length, doubling time, and onset of stationary phase to cells (Fig. 2 and 4). The observations that cells with increased *ddrR* expression grew slower than WT cells, while both *ddrR* insertion and *ddrR E14X* nonsense mutants grew faster (Fig. 4), supported our hypothesis that the DdrR protein possessed CDI properties.

Two supplemental experiments suggested that *ddrR* was the only UmuDAb-DdrR-repressed gene whose actions affected CDI. Cells became filamented when possessing either a promoter mutation that decreases UmuDAb binding to the *ddrR–umuDAb* operator (27) or encoding a UmuDAb HTH DNA-binding mutant that increases expression of *ddrR*, e.g., by 165-fold (14) (Fig. S2). This HTH mutant also increases expression 92–263-fold (14) of two functional *umuDC* operons (56), but mutants in these genes [*umuD* (*A1S_0636*) and *rumB* (*A1S_1173*)] still showed WT-like cell filamentation after DNA damage (Fig. S3).

The mid-cell bulging seen in the Δ*umuDAb* mutant after DNA damage (Fig. 1A) supports a role for DdrR in these cells’ DDR. This morphology is different from that seen for the untreated *mrcA* (PBP1A) mutant (58) or MMC-treated *aciT* mutants in *A. baumannii* (59) or other *Acinetobacter* species (26), and may represent dysregulation of additional cell division genes or a different mechanism of action than AciT. AciT is a previously identified contributor to cell filamentation after ciprofloxacin treatment in *A. baumannii* (59) that was recently found to have CDI properties in *Acinetobacter* species (26) not part of the *A. calcoaceticus–A. baumannii* complex (60). It is interesting that the *ddrR*::Tn*LK* strain was significantly impaired in forming filaments after DNA damage, relative to WT (Fig. 3), as is an *aciT* mutant after either ciprofloxacin (59) or MMC (26) treatment. The *aciT* finding was proposed to point to an additional AciT-dependent mechanism of CDI (26), which is compatible with our findings about DdrR action as a CDI.

That a Δ*umuDAb* mutant still responded to DNA damage with filamentation (Fig. 1A) may be explained by the additional level of regulation conferred by the binding of EppR, a second repressor in the *A. baumannii* DDR, to the shared *umuDAb–ddrR* operator (25). Although the regulation of *ddrR* by EppR was not examined in that work, EppR binds less tightly to this operator than UmuDAb does, and hence some modest repression of *ddrR* by EppR may likely still occur in a Δ*umuDAb* mutant. We hypothesize that DNA damage would relieve EppR repression of *ddrR*, resulting in the additional filamentation seen in Fig. 1A. These investigations of EppR did not examine cell length phenotypes, so it is unknown how any *eppR* mutation would affect this phenotype. However, we predict that an *eppR* mutant would exhibit modest cell filamentation in basal growth conditions and that an *eppR*/*ddrR* double mutant would display WT-like cell lengths, as our Δ*umuDAb* Δ*ddrR* double mutant did.

Our transcriptome studies show that DdrR mediates repression of multiple cell division genes that facilitate cellular elongation, septal formation, and daughter cell separation as part of the normal cell cycle (40). These include the essential gene *advA* (36, 61); *advA*-depleted cells grow poorly and display filamentous growth and hypersusceptibility to fluoroquinolone and β-lactam antibiotics that target DNA and cell wall functions, respectively. Its action appears to be by participating in the Rod system for peptidoglycan production and elongation of cells during chromosome segregation. Although AdvA localizes to the mid-cell of dividing cells (36, 61), its role there is unknown. One study suggests that loss of AdvA expression over time causes mistargeting of divisome proteins ZipA, FtsN, B, K, and L, interacting with FtsN and ZipA in BACTH two-hybrid studies (62). DdrR repression of AdvA could be a means to affect cell division inhibition by a currently unknown mechanism.

Taken collectively, our results are compatible with, and add to, these studies of cell division inhibition in *Acinetobacter*, most notably the observation that both our *ddrR* mutants and an *aciT* mutant showed decreased filamentation after DNA damage, which is consistent with a different mechanism of CDI by DdrR. We previously observed that *umuDAb* is required for *aciT* repression under non-DNA-damaging growth conditions (40), which, by increasing *ddrR* expression, could contribute to the inhibition of cell division in this pathogen. That DdrR co-represses SOS genes by binding to one repressor, UmuDAb (14, 39), also raises the possibility that DdrR might affect *aciT* expression by interacting with another repressor besides UmuDAb and so cause CDI. Alternatively, as UmuDAb represses DdrR, DdrR could be the putative unknown protein that might interact with AciT to cause cell filamentation, as recently proposed (26). Both we and Ellison et al. (26) observed filamentation of Δ*umuDAb* mutant strains in different species, but to different extents. This result could reflect the use of rich LB media in their study vs the more restrictive minimal medium we used, whichthat may have resulted in the greater length of our Δ*umuDAb* mutant strain. Other small differences between data sets may be attributable to our experiments being conducted on different *Acinetobacter* species that contain different genomes, and different experimental protocols, e.g., the concentration of MMC used to induce the DDR was up to 10-fold higher in those studies (26).

Cell filamentation is a well-known phenomenon of inhibition of cell division that occurs after various forms of DNA damage and stress (63). However, cell division can be inhibited by a variety of mechanisms. For most Gammaproteobacteria, this action depends on SOS induction of the cytosolic CDI protein SulA, which directly inhibits formation of the Z-ring needed for cell division. However, Gram-positive species that lack SulA often utilize membrane-interacting CDIs (43), such as YneA or SosA, that bind FtsW to prevent septal peptidoglycan synthesis (44, 64), but not Z-ring formation. Similarly, the α-proteobacterium *C. crescentus* has two differentially regulated CDI proteins that also seem to act at late stages of cell division. The SOS-dependent CDI SidA binds to FtsW (42), while the SOS-independent CDI protein DidA, induced by DriD activator, binds to FtsN (65).

There are multiple mechanisms by which *Acinetobacter* species regulate their responses to DNA damage: UmuDAb and DdrR repress the error-prone polymerases by which drug resistance can arise (29, 66), while a WYL family activator, DdaA, induces another regulon of DDR genes (23), including *aciT* (26), as well as several prophages present in *A. baumannii* strains (67). Unsurprisingly, given these significant differences from the canonical, enteric-derived (LexA- and SulA-dependent) models, there are also multiple mechanisms by which *Acinetobacter* species regulate and inhibit cell division after DNA damage. Neither DdrR nor AciT perfectly matches the characteristics of the small (~77–105 AA) Gram-positive CDIs, as AciT is a 697 AA transmembrane protein (59), while DdrR is a small 76–78 AA protein predicted to be cytosolic, lacking a transmembrane domain (e.g., by TMHMM 2.0 [68] or CCTOP [69]) or any other protein domain. Establishing whether DdrR CDI actions occur by localizing to the mid-cell plane like SulA, interacting with the cell membrane like Gram-positive CDIs, or via regulating cell-division genes, such as *advA* and *aciT*, will help reveal important details about the diverse CDI responses in the DDR of non-*Enterobacteriaceae* Gram-negative bacteria and so build a new model of these critical control responses to DNA damage.

The multidrug-resistance problem in *A. baumannii* and other ESKAPE organisms (30) and the paucity of new antibiotics in research pipelines emphasize our need for diverse treatment approaches, such as targeting LexA action to potentiate antibiotic action (49). It is thus crucial to discover how pathogens lacking common Gram-negative cell division proteins but possessing unique ones perform CDI. This might allow structure-based design of inhibitors of protein interactions needed for cell division.

## SUPPLEMENTAL MATERIAL

Supplemental figures jb.00416-26-s0001.pdf Fig. S1 to S3.

ASM does not own the copyrights to Supplemental Material that may be linked to, or accessed through, an article. The authors have granted ASM a non-exclusive, world-wide license to publish the Supplemental Material files. Please contact the corresponding author directly for reuse.
