# Supplementary material for: DdrR acts as a DNA damage-induced cell division inhibitor in Acinetobacter baumannii
Source: J Bacteriol. 2026 Sep 9;208(10):e00416-26. doi: 10.1128/jb.00416-26 (PMC13689564; doi:10.1128/jb.00416-26)
Supplement: Supplementary file 1 [file jb.00416-26-s0001.pdf]

Supplemental data

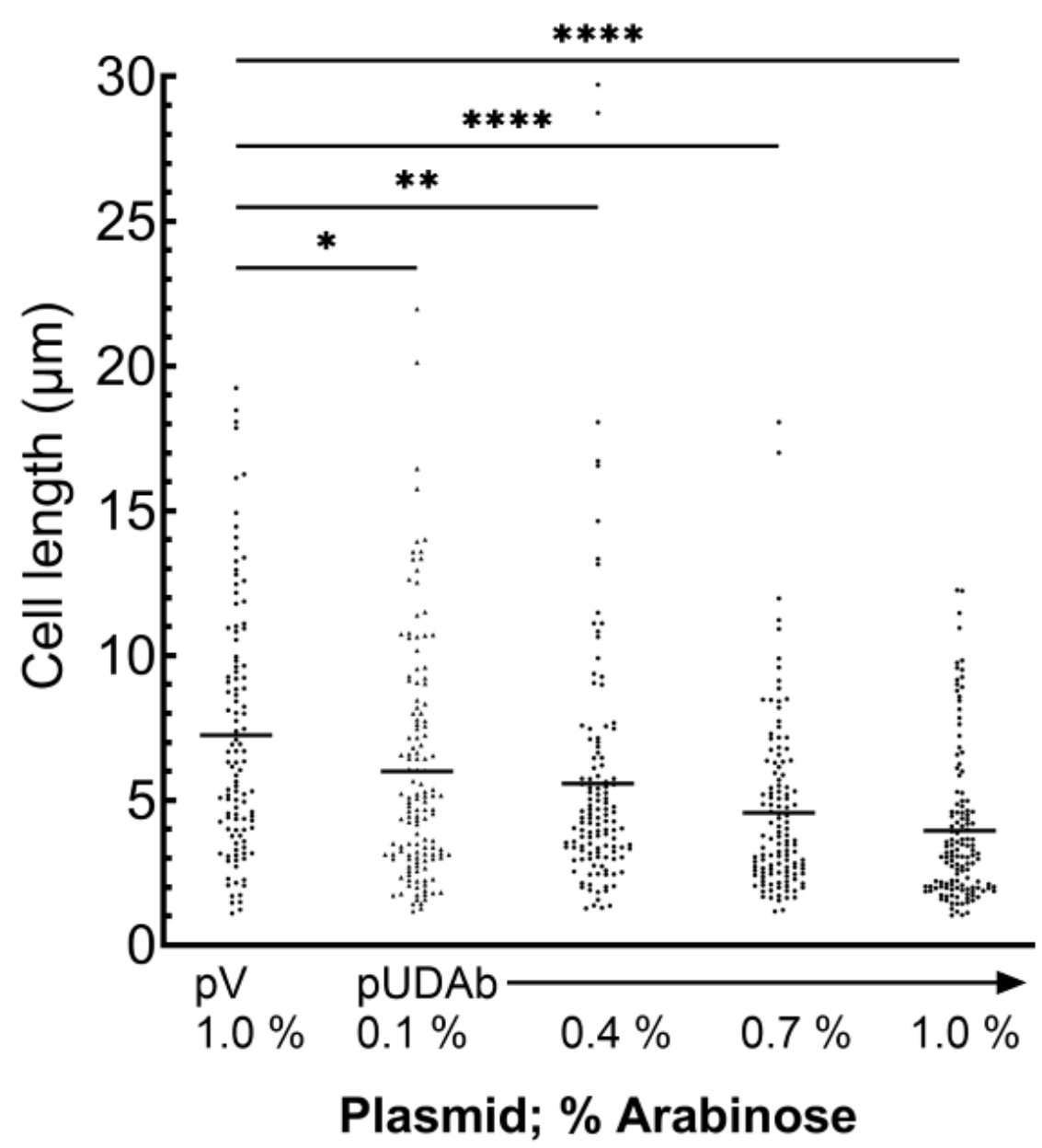

Fig S1. Cell lengths of a null Δ*umuDAb* mutant of *A. baumannii* ATCC 17978 impacted by *umuDAb* complementation. The Δ*umuDAb* (strain JH1600) cells carried the arabinose-inducible vector pVRL2 either containing no insert ("pV") or expressing UmuDAb ("pUDAb") to complement the Δ*umuDAb* mutation. Cells were grown in varying percent concentrations (ranging from 0.1 – 1 %) of arabinose in LB medium plus gentamicin for 3 h. Cell lengths were then measured with MicrobeJ (n = 109-133) after staining with crystal violet. Horizontal bars represent the mean. Pairwise comparison bars represent one-way ANOVA with Dunnett's multiple comparisons test. **** =P < 0.0001; *** =P < 0.001; ** =P < 0.01; * =P < 0.05. ns, not significant.

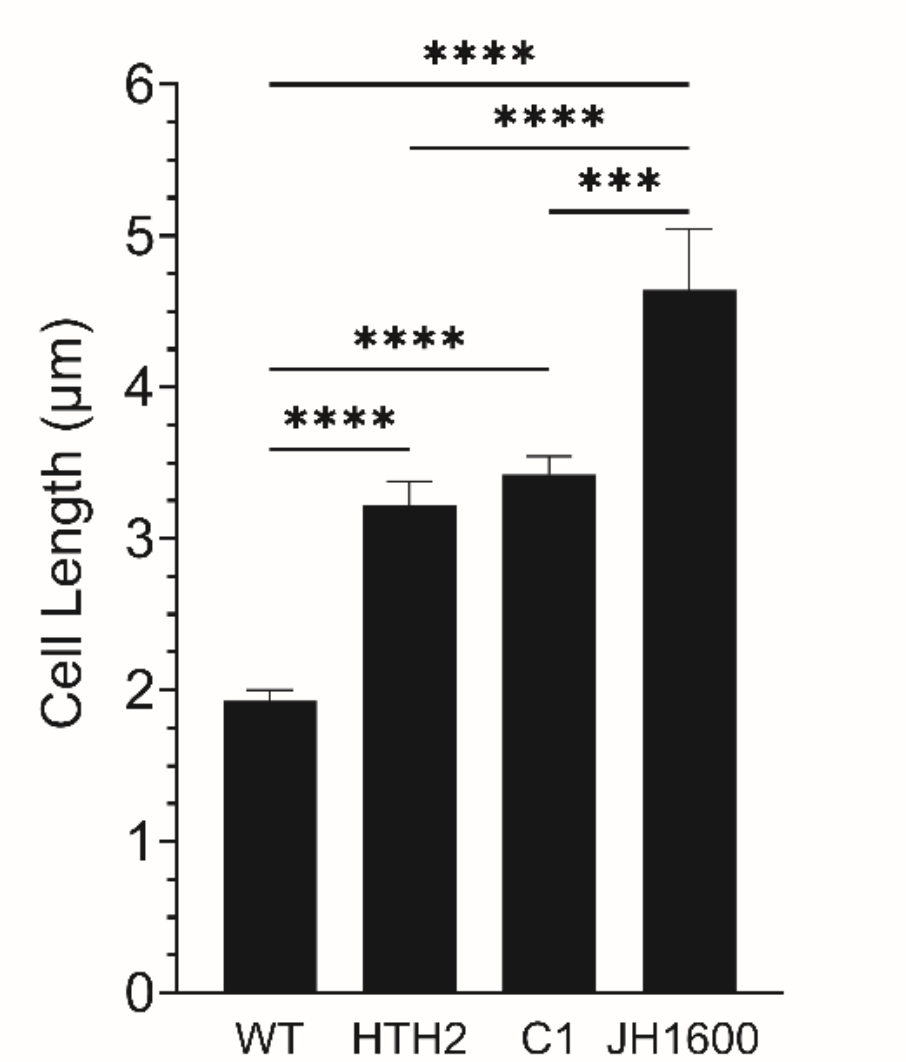

Fig S2. Cell lengths in *A. baumannii* ATCC 17978 and in strains possessing mutations in either the UmuDAb repressor or the joint *ddrR*-*umuDAb* operator. Shown are lengths of cells grown in LB broth. Strains contain the following mutations: HTH2 = mutation in the helix-turn-helix motif of UmuDAb that increases *ddrR* and *umuDAb* expression ≥165-fold in strain MF2 (14); C1 = *ddrRp1* mutation in the shared *ddrR* and *umuDAb* UmuDAb binding operator site that reduces UmuDAb binding by ~40 % (27) in strain DC11; JH1600 has a Δ*umuDAb* mutation with *umuDAb* replaced by a kanamycin resistance cassette (34). Pairwise comparisons represent ANOVA mixed-effects analysis with Dunnett's multiple comparison tests. **** =$P < 0.0001$; *** =$P < 0.001$; ** =$P < 0.01$; * =$P < 0.05$. ns, not significant. Error bars represent the standard deviation of the mean.

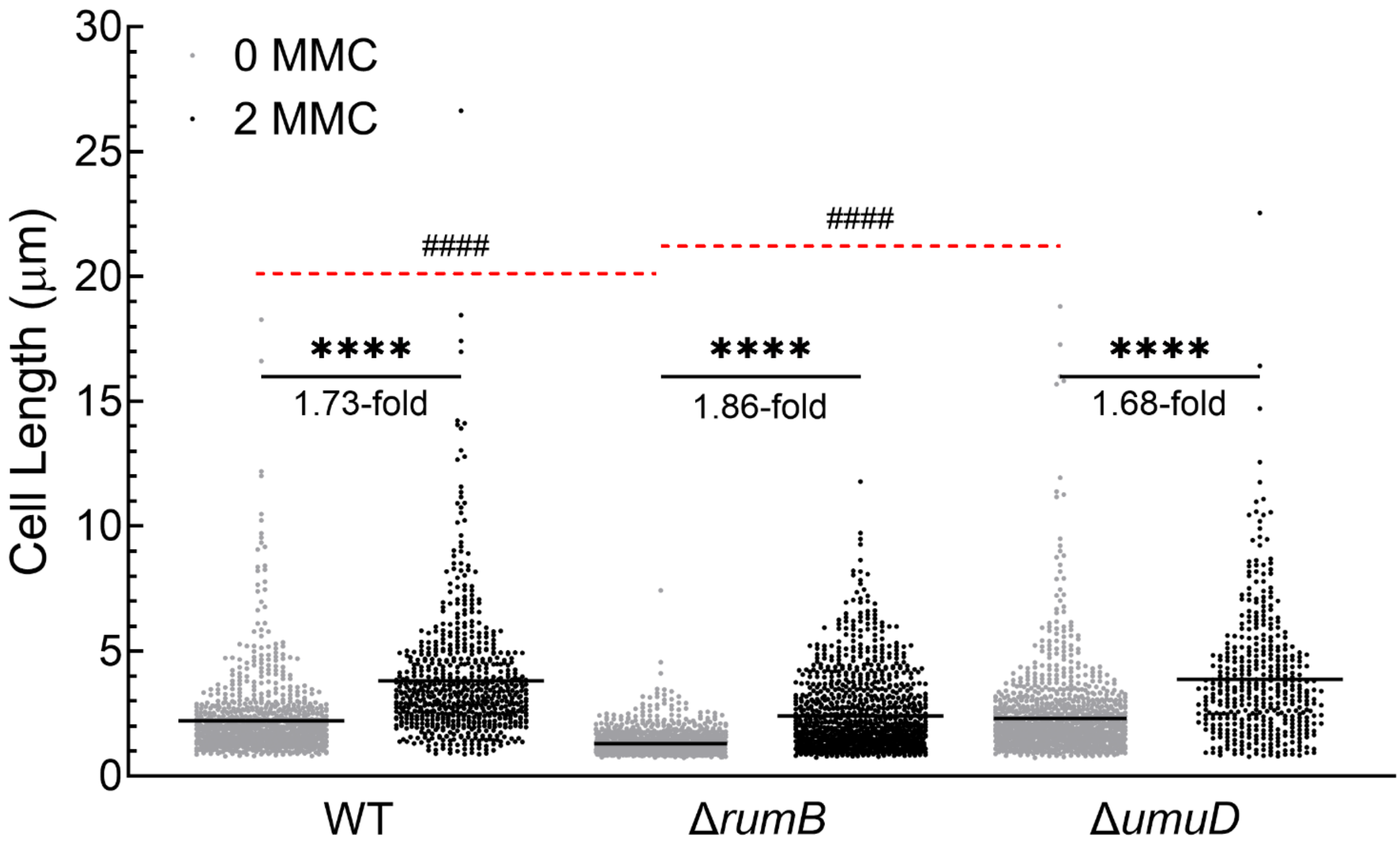

Fig S3. Cell lengths of WT and error-prone polymerase Δ*rumB* and Δ*umuD* mutant strains before and after DNA damage (MMC treatment). Data shown are of six biological replicates each of the WT, Δ*rumB*::Ω (deletion of *A1S_1173*), and Δ*umuD*::*kanR* (deletion of *A1S_0636*) strains of *A. baumannii* ATCC 17978 in minimal media 3 h after being left untreated or exposed to 2 μg/mL MMC (gray dots, untreated; black dots, exposed). The ratio of treated to untreated cell lengths was the same in all strains (ANOVA with Tukey's multiple comparison) although the Δ*rumB* Δ *umuC* mutant was shorter than the WT and Δ*umuD* mutant strains under untreated conditions (see "#" symbols above red dashed comparison bars). Cell lengths were measured with MicrobeJ after staining with crystal violet. n = 427-1162; GraphPad 'expanded view' used to display the large dataset. Horizontal bars represent the mean. Pairwise comparison bars represent one-way ANOVA and Tukey multiple comparison tests. **** =*P* < 0.0001; *** =*P* < 0.001; ** =*P* < 0.01; * =*P* < 0.05. ns, not significant.
